# Supplementary figures and images for: Longitudinal Characterization of Immune Response in a Cohort of Children Hospitalized with Multisystem Inflammatory Syndrome
Source: Children (Basel). 2023 Jun 16;10(6):1069. doi: 10.3390/children10061069 (PMC10297301; doi:10.3390/children10061069)

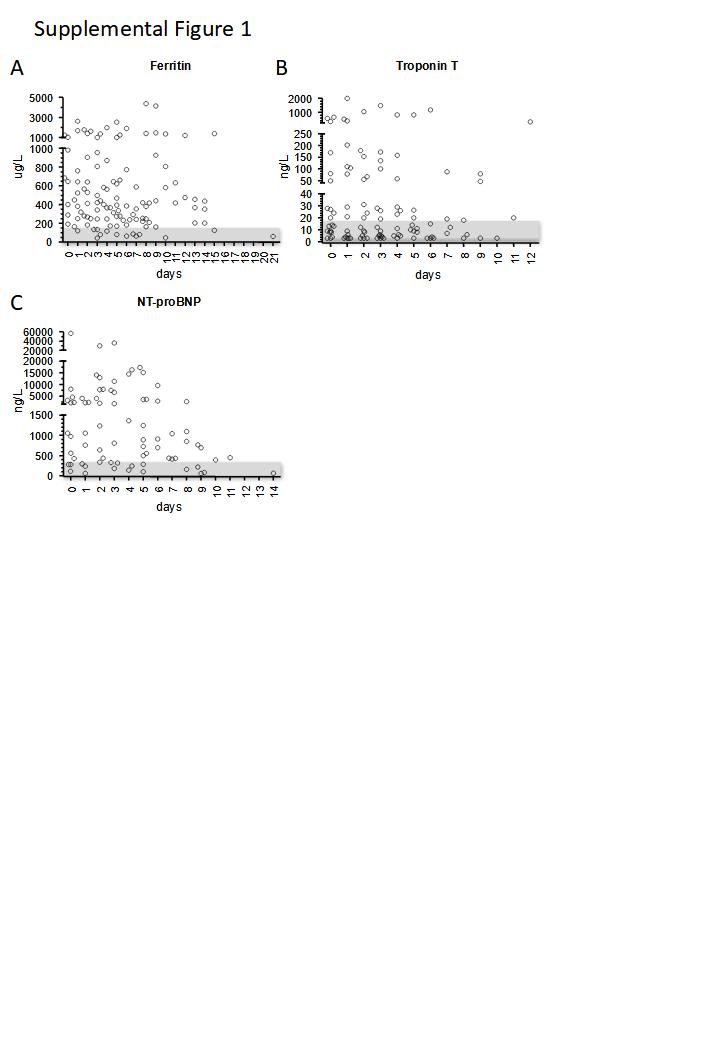

Supplement: Supplementary file 1 [file children-10-01069-s001.zip › Supplemental Figure S1.jpg]

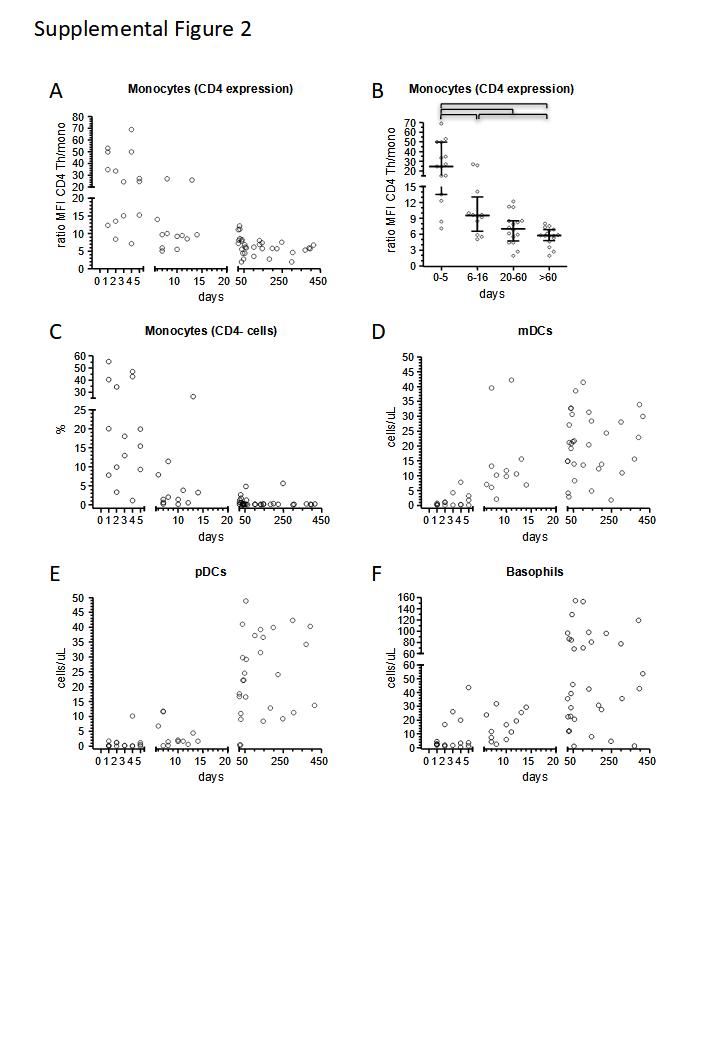

Supplement: Supplementary file 1 [file children-10-01069-s001.zip › Supplemental Figure S2.jpg]

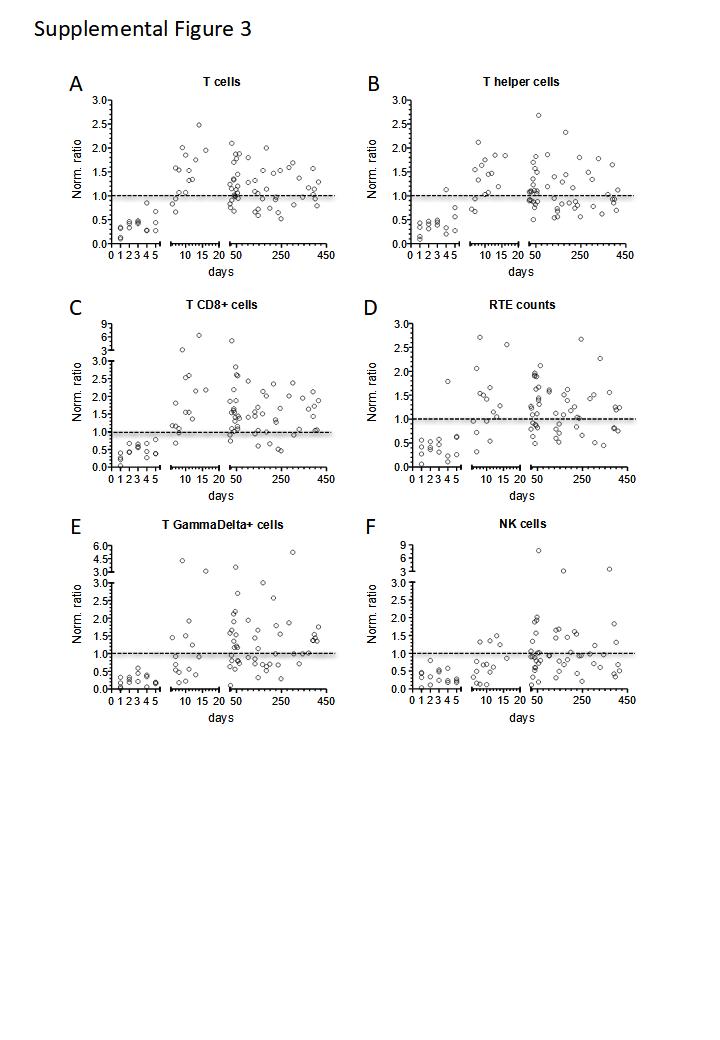

Supplement: Supplementary file 1 [file children-10-01069-s001.zip › Supplemental Figure S3.jpg]

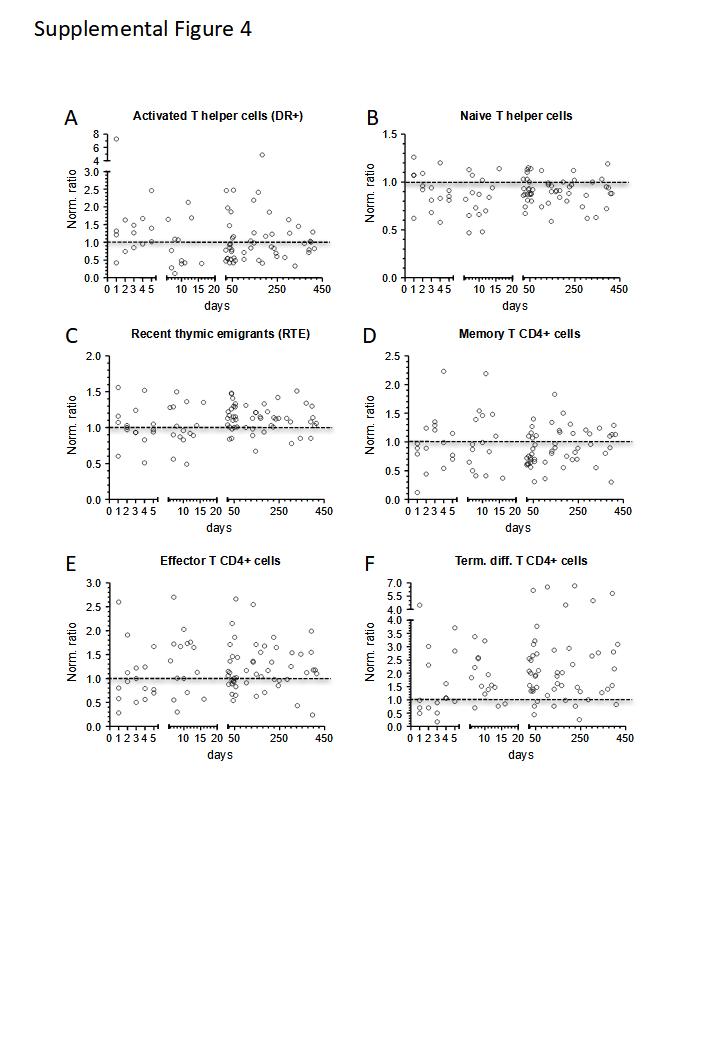

Supplement: Supplementary file 1 [file children-10-01069-s001.zip › Supplemental Figure S4.jpg]

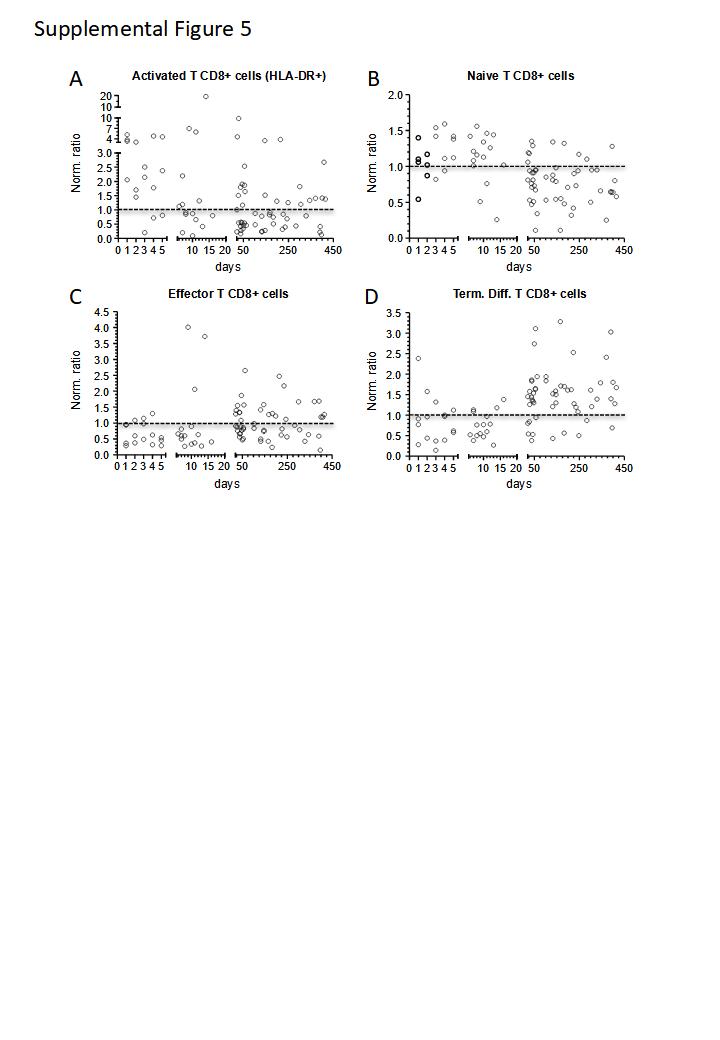

Supplement: Supplementary file 1 [file children-10-01069-s001.zip › Supplemental Figure S5.jpg]

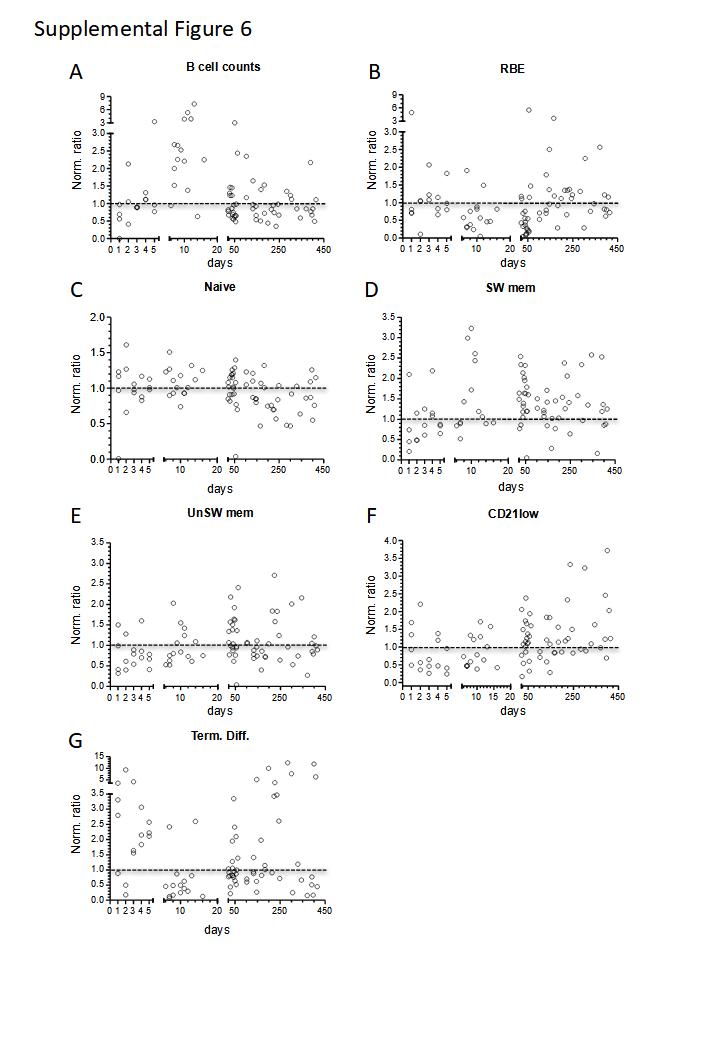

Supplement: Supplementary file 1 [file children-10-01069-s001.zip › Supplemental Figure S6.jpg]
